# SARS-CoV-2 pre-exposure prophylaxis with tixagevimab/cilgavimab (AZD7442) provides protection in inborn errors of immunity with antibody defects: a real-world experience

**DOI:** 10.3389/fimmu.2023.1249462

**Published:** 2023-10-26

**Authors:** Federica Pulvirenti, Giulia Garzi, Cinzia Milito, Eleonora Sculco, Maddalena Sciannamea, Anna Napoli, Lilia Cinti, Piergiorgio Roberto, Alessandra Punziano, Maria Carrabba, Eva Piano Mortari, Rita Carsetti, Guido Antonelli, Isabella Quinti

**Affiliations:** ^1^ Reference Centre for Primary Immune Deficiencies, Sapienza University Hospital “Policlinico Umberto I”, Rome, Italy; ^2^ Department of Molecular Medicine, Sapienza University, Rome, Italy; ^3^ Microbiology and Virology Unit, Sapienza University Hospital “Policlinico Umberto I”, Rome, Italy; ^4^ Department of Translational Medical Sciences, University of Naples Federico II, Naples, Italy; ^5^ Department of Internal Medicine, Fondazione IRCCS Ca' Granda Ospedale Maggiore Policlinico, Milan, Italy; ^6^ B Cell Unit, Immunology Research Area, Bambino Gesù Children's Hospital, Istituto di Ricovero e Cura a Carattere Scientifico (IRCCS), Rome, Italy

**Keywords:** SARS-CoV-2, COVID-19, inborn errors of immunity, immunoglobulin replacement (IgRT), monoclonal antibody, tixagevimab/cilgavimab prophylaxis, common variable immune deficiency (CVID)

## Abstract

**Background:**

Preventive strategies against severe COVID-19 in Inborn Errors of Immunity (IEI) include bivalent vaccines, treatment with SARS-CoV-2 monoclonal antibodies (mAbs), early antiviral therapies, and pre-exposure prophylaxis (PrEP).

**Objective:**

To assess the effectiveness of the PrEP with tixagevimab/cilgavimab (AZD7442) in IEI with primary antibody defects during the COVID-19 Omicron wave.

**Methods:**

A six-month prospective study evaluated the SARS-CoV-2 infection rate and the COVID-19 severity in the AZD7442 group, in the no-AZD7442 group, and in a group of patients with a recent SARS-CoV-2 infection (< three months). Spike-specific IgG levels were measured at regular intervals.

**Results:**

Six out of thirty-three patients (18%) and 54/170 patients (32%) became infected in the AZD7442 group and in the no-AZD7442 group, respectively. Within 90 days post-administration, the AZD7442 group was 85% less likely to be infected and 82% less likely to have a symptomatic disease than the no-AZD7442 group. This effect was lost thereafter. In the entire cohort, no mortality/hospitalisation was observed. The control group of 35 recently infected patients was 88% and 92% less likely to be infected than the AZD7442 and no-AZD7442 groups. Serum anti-Spike IgG reached the highest peak seven days post-AZD7442 PrEP then decreased, remaining over 1000 BAU/mL 180 days thereafter.

**Conclusion:**

In patients with IEI and antibody defects, AZD7442 prophylaxis had a transient protective effect, possibly lost possibly because of the appearance of new variants. However, PrEP with newer mAbs might still represent a feasible preventive strategy in the future in this population.

## Introduction

Cohort studies in patients with Inborn Errors of Immunity (IEI) with predominant antibody defects showed a mild COVID-19 course in most patients (1–5). Deaths were often due to respiratory failure in those with preexisting end-stage lung disease (6–8). Pandemic mitigation policies to protect patients with IEI have changed over time (6). In the first year, COVID-19 prevention strategies were mainly based on social distancing measures (7). As the COVID-19 pandemic evolved, significant progress has been made with the availability of vaccines, monoclonal antibodies (mAbs) targeting the SARS-CoV-2 spike protein, and antiviral drugs (7). Immunization against SARS-CoV-2 has represented the cornerstone strategy, with current recommendations including the primary immunization cycle followed by booster doses (8). Like many countries, Italy encouraged booster doses for high-risk populations, including people with IEI (9). Published data on IEI with antibody deficiency indicated a suboptimal antibody response after completing the first vaccine cycle (10) with a lower magnitude of antibody response and a reduced virus-neutralizing function compared to healthy controls (11–16). In addition to immunisation strategies, in the second year of the pandemic, we took advantage of the newly developed anti-Spike protein mAbs for early COVID-19 treatment (1, 17, 18), with a protective effect in reducing hospitalisation and severe outcome across the alpha, delta and early omicron waves (17).

From July 2022 onwards, this protection was decreased due to the emergence of new variants of concern (VOC), including Omicron BA4/BA5 towards which vaccines and mAbs were less efficient and had lower neutralising power (19, 20). Due to the changing SARS-CoV-2 trajectory, the preventive strategy in vulnerable patients had to be updated, including bivalent vaccines, early therapies, and, more recently, mAbs given as pre-exposure prophylaxis (PrEP). Two long-acting monoclonal antibodies, tixagevimab/cilgavimab were combined to produce AZD7442 (Evusheld®) (21). In December 2021, AZD7442 was authorised as PrEP against COVID-19 for patients over 12 years with moderate to severely compromised immunity (22). Data from two phase-III trials demonstrated that, compared to placebo, AZD7442 recipients had an 83% and 51% relative risk reduction in developing symptomatic and severe COVID-19 diseases at a median follow-up of 6 months, respectively (21, 23). In a real-life study on a heterogeneous cohort of immunocompromised subjects, the AZD7442-treated group was 50% less likely to become infected and 92% less likely to be hospitalised/die than those not-administered AZD7442 (24).

Here, we aimed to assess the efficacy of PrEP with AZD7442 in the prevention of SARS-CoV-2 infection, COVID-19 severity, and mortality during the Omicron-predominant SARS-CoV-2 infection outbreak in IEI patients with primary antibody defects (25).

## Materials and methods

### Study design

A six-month prospective-observational study was conducted from July 1 to December 31, 2022. We screened 238 IEI patients >18 years regularly followed up in the Care Centers in Rome (Policlinico Umberto I, Sapienza University), Naples (Federico II University), and Milan (Ospedale Maggiore Policlinico, University of Milan), Italy. The population study included subjects with primary antibody deficiency diagnosed according to the ESID criteria (26). All participants were treated with immunoglobulin replacement therapy (IgRT) by intravenous (IVIG), subcutaneous (SCIG), or facilitated immunoglobulins (fSCIG). Booster doses of BNT162b2 mRNA vaccine were offered to the entire IEI cohort in September 2021, six months after completing the primary immunization cycle, and in April 2022. From June 2022, AZD7442 (300 mg: 150 mg tixagevimab/150 mg cilgavimab) was offered as PrEP for SARS-COV-2 infection to patients with a minimum weight of 40 kg who did not have a positive test result (PCR or antigen) in the previous month, and who were not vaccinated against COVID-19 in the previous two weeks. Eligible patients received an SMS and an email advising they were suitable for PrEP and inviting them to contact their IEI centre to make an appointment for AZD7442 administration. If no appointment was made within 15 days, an SMS and an email were sent again. Clinicians provided informed consent on the treatment, effectiveness, and contraindications. AZD7442 was administered at least one week after the IgRT administration.

**Figure 1:**
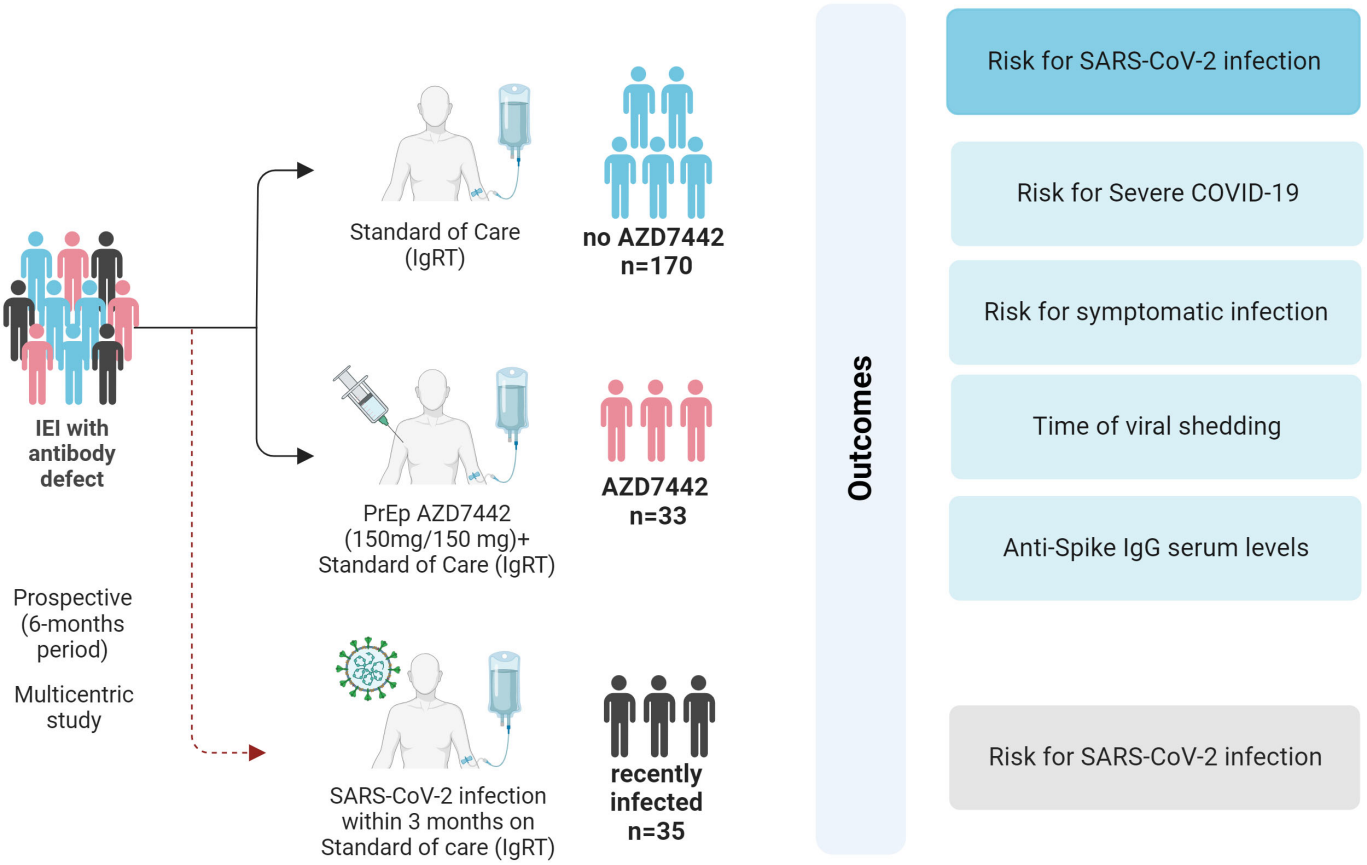
Study design and outcomes. SARS-CoV-2 infection and COVID-19 severity were evaluated in the AZD7442 group (33 patients), the no-AZD7442 group (170 patients) and in patients with a recent SARS-CoV-2 infection (35 patients). COVID-19, coronavirus-19 disease; IEI, inborn errors of immunity; IgRT, immunoglobulin replacement therapy; IgG, immunoglobulin G; n, number; PrEP, pre-exposure prophylaxis; SARS-CoV2, Severe acute respiratory syndrome coronavirus 2.

Enrolment was allowed between July 1, 2022, and August 31, 2022. The study population was divided into two groups: 33 patients received AZD7442 as PrEP (AZD7442 group), and 170 patients did not receive AZD7442 (no-AZD7442) group (either did not read the SMS/email, or declined to receive AZD7442, or did not take steps to attend an appointment for whatever reason). Data from 35 patients excluded from enrolment as being positive for SARS-CoV-2 in the previous three months were separately analysed (recently infected group) (**Figure 1**). For the AZD7442 group, we used the date of administration of tixagevimab/cilgavimab to identify the date of entry into the study; for the no-AZD7442 group, we used the date of the signature of the informed consent as the date of entry into the study. For the recently infected group, July 1st was considered the date of entry into the study. All participants who agreed to participate signed the written informed consent form at enrolment.

After enrolment, clinicians reminded all patients of prevention measures and on the need to contact the hospital in case of SARS-CoV-2 infection. Patients were tested for SARS-CoV-2 by PCR or by nasopharyngeal swab antigen detection every time they attended a hospital site (i.e., to receive IgRT), in case of positive household contacts irrespective of symptoms and upon onset of symptoms possibly related to COVID-19. During the study period, we interviewed the participants monthly via in-person visits or SMS/emails to verify if an infection had occurred from the last observation. All participants were allowed to continue their treatments, including IgRT as standard therapy for the underlying antibody deficiency. In the case of SARS-CoV-2 infection, specific treatments were prescribed by clinicians based on the clinical assessment and ongoing recommendation.

At enrolment, demographic and IEI-related health issues were collected to allow comparison of outcome measures, adjusting for differences between groups. Health factors included IEI diagnosis, immunoglobulins serum levels, lymphocyte count, and IEI-related comorbidities (Chronic Obstructive Pulmonary Disease (COPD) (27), malignancies, autoimmune manifestations, and granulomatous diseases), immunisation status, and number of doses of BNT162b2 mRNA vaccine administered. We defined IEI with a complicated phenotype as having at least one of the non-infective IEI-related comorbidities (25). The Ethical Committee of the Sapienza University of Rome (Prot. 0521/2020, July 13, 2020) approved the study. The study was performed following the Good Clinical Practice guidelines, the International Conference on Harmonization guidelines, and the most recent version of the Declaration of Helsinki.

### Study outcomes

The primary study outcome was SARS-CoV-2 infection, defined as any person with a positive PCR or positive antigen test result from 1st July 2022 – 31st December 2022. Secondary endpoints included: 1) severe COVID-19 disease; 2) symptomatic infection; 3) duration of the viral shedding; 4) additional SARS-CoV-2 specific treatments; 5) anti-Spike IgG serum levels.

COVID-19 symptoms severity was scored according to the World Health Organization stage. We defined severe COVID-19 disease as either COVID-19-related hospitalisation or all-cause mortality, assessed in each group for the same periods. In SARS–CoV–2 positive patients, we calculated the duration of viral shedding by recording the dates of the first positive and the first negative nasopharyngeal swab. In the AZD7442 group, we measured the anti-Spike IgG serum levels at enrolment (T0), seven days (T7), 120 days (T120), 150 days (T150), and 180 days (T180) after PrEP administration. We collected samples for anti-Spike IgG levels on the day of the IgRT administration, just before immunoglobulins administration.

### SARS-CoV-2 anti-Spike antibodies

We evaluated SARS-CoV-2 spike S1/S2 protein-specific IgG antibody levels by the DiaSorin Liaison SARS-CoV-2 Trimeric S IgG chemiluminescence immunoassay (CLIA) (DiaSorin S.p.A, Saluggia, VC, Italy). We reported the sensitivity and specificity performance according to the manufacturer’s instructions. We expressed the levels of anti-SARS-CoV-2 IgG antibodies using the World Health Organization International Standard (NIBSC code. 20/268) binding antibody unit (BAU/mL). The assay’s lower and upper detection limits were 4.81 BAU/mL and 2080 BAU/mL, respectively. Samples testing >2080 BAU/mL were further diluted.

### B-cell responses to SARS-CoV-2 immunization

We studied the B-cell responses to SARS-CoV-2 immunisation with the BNT162b mRNA vaccine in a subgroup of 78 patients randomly selected over the participants. Blood samples were obtained ten days after a booster dose administration. We isolated the peripheral blood mononuclear cells (PBMCs) by Ficoll Paque™Plus 206 (Amersham PharmaciaBiotech) density gradient centrifugation and immediately froze and stored in liquid nitrogen until use. The freezing medium contained 90% Fetal Bovine Serum (FBS) and 10% DMSO. To detect SARS-CoV-2 specific B cells, a biotinylated protein spike was individually multimerized with fluorescently labelled streptavidin as previously described (28). Briefly, to identify the Spike-specific memory B cells (MBC), we used two different streptavidin-conjugated fluorochromes, one with a very high brightness index (PE) and the other with a moderate brightness index (BUV395), in order to be able to distinguish low-affinity MBCs (only visible with a very bright fluorochrome such as PE) from high-affinity MBCs (double positive for PE and BUV395). We defined the spike-specific MBCs as low-affinity (positive for PE, S+) or high-affinity (double positive for PE and BUV395, S++). We acquired stained PBMC samples on FACs LSRFortessa (BD Bioscience). At least 2x106 cells were acquired and analysed using Flow-Jo10.8.1 (BD Bioscience). We only performed phenotype analysis of antigen-specific B cells in subjects with at least ten cells detected in the respective antigen-specific gate. The gating strategy is shown in **Supplementary Figure 1A**.

### Statistical analysis

The primary analysis was to investigate the risk for SARS-CoV-2 infection in the AZD7442 vs. the no-AZD7442 group. We described continuous variables using median and interquartile ranges (IQR) and categorical variables using frequencies and percentages. Comparisons of continuous parameters between groups were calculated with a t-test if normally distributed (as tested by the Kruskal-Wallis’s test) and with a Mann-Whitney U test if not normally distributed; differences in frequencies between groups were calculated by using the χ2 exact test. To assess the relationship between AZD7442 administration status and outcome variables over time, we used the Kaplan-Meier product-limit estimates and based on a Log-rank of the difference between the two treatment groups at the time of the SARS-CoV-2 infection, with no adjustment for baseline covariates. The difference between the two treatment groups was calculated monthly from the enrolment until the end of the study period. In the no-AZD7442 group, we compared specific anti-Spike IgG serum levels at different time points over the study period. Values were compared by the nonparametric Kruskal-Wallis’s test, and if not significant, the Wilcoxon matched-pair signed-rank test or the two-tailed Mann-Whitney U test was used. Differences were significant when p was less than 0.05. Analyses were carried out using SPSS software, version 18 (SPSS, Chicago, IL).

## Results

### Patients

Of 238 IEI candidates, 33 patients (16%) were treated with 300-mg AZD7442 (tixagevimab/cilgavimab, 150 mg/1.5 mL and 150 mg/1.5 mL) and 170 patients (84%) did not take steps to attend an appointment for whatever reason or declined the treatment. The AZD7442 administration was well tolerated, with no patient reporting major adverse events. When comparing variables considered as potential confounders (**Table 1**), the AZD7442 group had lower serum IgA levels (p=0.006) and lower post-booster anti-Spike IgG serum levels (p=0.010, **Table 2**), suggesting a more severe immune defect in those who agreed to receive PrEP. Age, gender, complicated phenotype, total lymphocyte, lymphocyte subsets count, number and timing of previous vaccine doses were comparable in the two groups (**Table 1**). Thirty-five patients (age 54 years, IQR: 41-66) who tested positive for SARS-CoV-2 in the three months before the enrolment were considered as controls and included in the recently infected group. Characteristics of recently infected patients and comparisons with the study groups are reported in **Supplementary Table 1**.

**Table 1:** Baseline characteristics of the enrolled IEIs population.

Characteristics	no-AZD7442 n= 170	AZD7442 n=33	p-value
Age (years), median (IQR)	51 (39-60)	55 (42-66)	0.671
Gender (female), n (%)	84 (49.7)	17 (51.5)	0.849
IgA (mg/dL), median (IQR)	5.0 (2-22)	2.0 (0-2)	0.006
IgG* (mg/dL), median (IQR)	651 (602-701)	645 (608-695)	0.542
IEI diagnosis			
CVID, n (%)	132 (77.6)	27 (81.8)	0.595
XLA, n (%)	8 (4.7)	1 (3.0)	0.669
Good Syndrome, n (%)	4 (2.4)	1 (3.0)	0.818
UAD, n (%)	14 (8.2)	3 (9.1)	0.871
Others n (%)	12 (7.1)	1 (3.0)	0.387
Lymphocytes count, median (IQR)	1490 (1123-1950)	1240 (910-1590)	0.100
CD3+CD4+ (cell/mm3), median (IQR)	519 (400-727)	951 (332-1328)	0.287
CD19+ (cell/mm3, median (IQR)	57.1 (19.8-148)	43.9 (0-124.9)	0.356
MBC (CD19+CD27+, % of B cells), median (IQR)	22.1 (10.5-39.8)	20.0 (4.5-41-2)	0.774
Complicated phenotype, n (%)	76 (45.0)	19 (57.6)	0.175
COPD, n (%)	57 (33.6)	15 (46.7)	0.190
Prior episode of COVID-19, n (%)	42 (24.7)	13 (39.4)	0.082
SARS-CoV-2 vaccine doses ≥3, n (%)	146 (86)	30 (91.3)	0.436
Last SARS-COV-2 vaccine dose within six months, n (%)	84 (49.5)	13 (38.5)	0.292

IEI, inborn errors of immunity; CVID, common variable immunodeficiency; XLA X, linked agammaglobulinemia; UAD, undefined antibody deficiency; COPD, Chronic Pulmonary Disease; COVID-19, coronavirus disease 2019; IQR, interquartile range; SARS-CoV-2, severe acute respiratory syndrome coronavirus *residual IgG serum levels.

**Table 2:** Post-immunization response in the AZD7442 and no-AZD7442 group after the third dose of the BNT162b2 vaccine.

Characteristics	Overall n=78	no-AZD7442# n=62	AZD7442# n= 16	HD	#p value
anti-Spike IgG (BAU/mL), median (IQR)	89.4 (7.3-625.3)	160.3 (8.7-657.3)	4.5 (0.5-111.0)	5120 (2656-7986)	0.010
S+ MBC (% of B cells), median (IQR)	0.06 (0.0-0.23)	0.0 (0-0.23)	0.13 (0.0- 0.18)	0.6 (0.4-1.0)	0.893
S++ MBC (% of B cells), median (IQR)	0 (0-0.03)	0.0 (0.0-0.05)	0.0 (0.0-0.0)	0.4 (0.2-0.7)	0.443
RBD+ B cells (% of S+MBCs), median (IQR)	0 (0-0.1)	0.0 (0.0-4.2)	0.0 (0.0-0.0)	28.6 (24.1-36.7)	0.379
CD19+ B cells (% of lymphocytes), median (IQR)	3.1 (2.0-4.9)	3.0 (1.9-4.8)	3.4 (3.0-7.3)	10.4 (5.8–13.4)	0.255
MBC (% of B cells), median (IQR)	22.4 (8-32.7)	22.6 (8.3-31.7)	21.9 (5.4-46.1)	31 (25.9-42.4)	0.858
Switched MBCs (% of MBCs), median (IQR)	5.9 (3.2-14.8)	6.5 (3.4-14.5)	5.0 (1.4-14.8)	12 (3.1–46.2)	0.462
SARS-CoV-2 infection post-immunization;					
non-responders/poor responders, n (%)	12/56 (21.4)	12/44 (27.3)	0/12 (0)		0.041
weak responders, n, (%)	4/22 (18.2)	4/18 (22.2)	0/4 (0)		0.541

Fisher’s test and Mann U Whitney test were performed as indicated to compare the AZD7442 and no-AZD7442 groups. For healthy donors (HD) values were obtained as reported by Piano Mortari et al. (27).

### Post-BNT162b2 immunization responses

**Figure 2:**
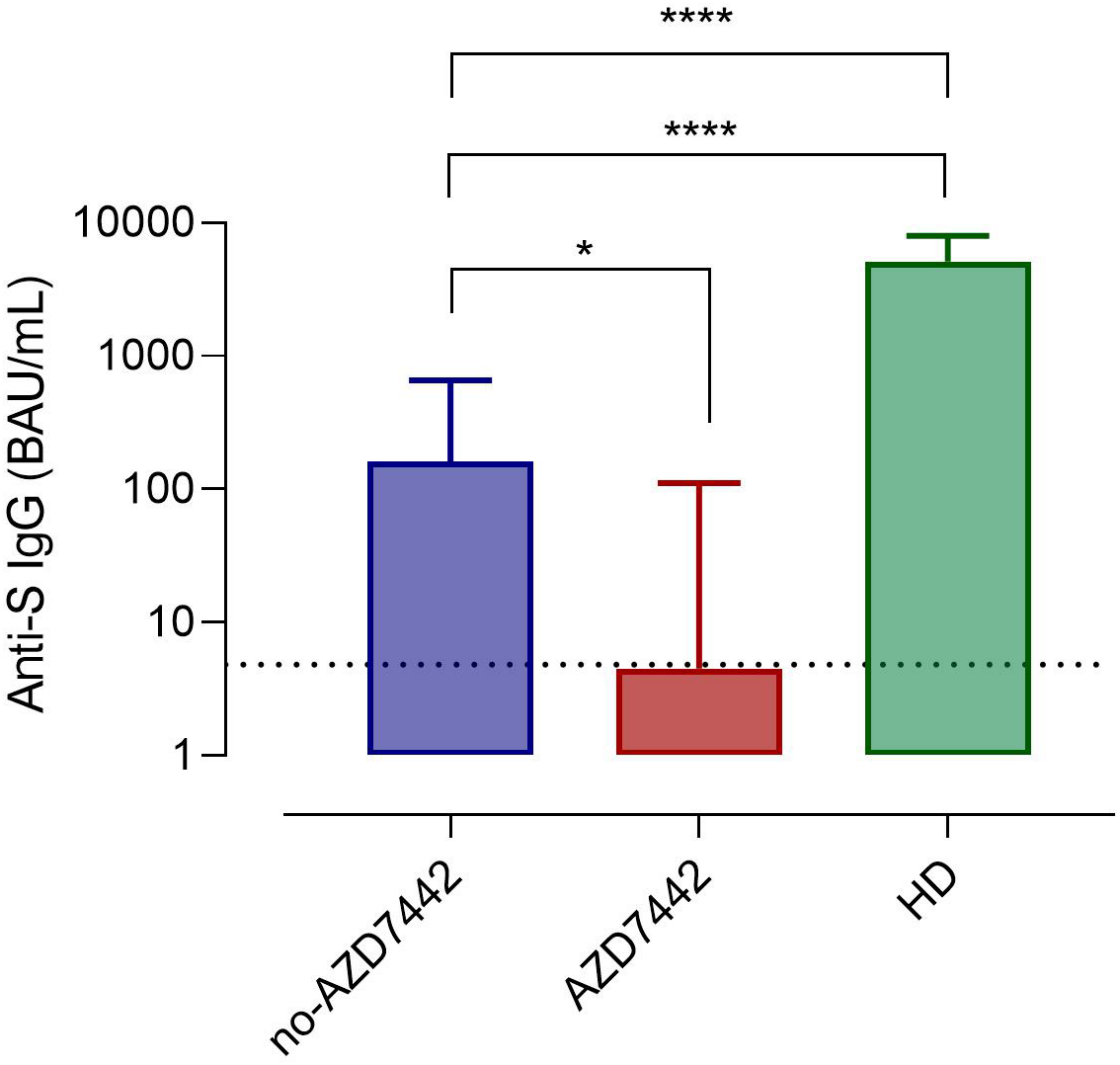
Anti-Spike IgG serum levels in patients in the AZD7442 group (red), in those in the no-AZD7442 group (blue) and in healthy controls (green). Bars indicate the median. The lower detection limit of the assay is represented by the dashed line. Non-parametric Mann–Whitney t-test was used to evaluate the statistical significance. Two-tailed P value significance is shown as * p<0.05, ****p<0.0001. anti-S IgG, anti-Spilke protein immunoglobulin G; BAU, binding antibody unit; HD, healthy controls.

During the study, 86% of patients were immunized with three or four doses of the BNT162b2 vaccine (**Table 1** and **Supplementary Table 1**). As previously shown (10, 28), the efficacy of SARS-CoV-2 immunisation is heterogeneous in IEI. Data on the production of anti-Spike IgG and specific MBC with low (S+) and high (S++) affinity, and Spike-specific RBD developed post-BNT162b2 booster were available for 78 participants, of which 16 were treated by PrEP. In our cohort, combining specific antibodies and specific B-cell responses, none of the patients produced a response comparable to what was observed in healthy subjects. Thirty-six percent of patients did not produce specific antibodies nor specific B cells (non-responders), 36% produced low levels of specific antibodies but did not generate specific high-affinity (S++) MBC and RBD+ B cells (poor responders), and 28% produced low levels of specific antibodies and generated low frequency of specific high-affinity MBC (weak responders) (**Table 2**). Post-immunization anti-SpikeIgG serum levels were lower in the AZD7442 group (median 4.5, IQR: 0.5-111.0) compared to the no-AZD7442 group (median 160.3, IQR 8.7-657.3, p=0,010) (**Figure 2**), suggesting a more severe immune-defect in those who agreed to receive PrEP. None was infected by SARS-CoV-2 in the AZD7442 group. In particular, none of the non-responders/poor-responders was infected in the AZD7442 group, whereas 27% were infected in the no-AZD7442 group (p=0.041) (**Table 2**).

### Risk of SARS-CoV-2 infection

**Figure 3:**
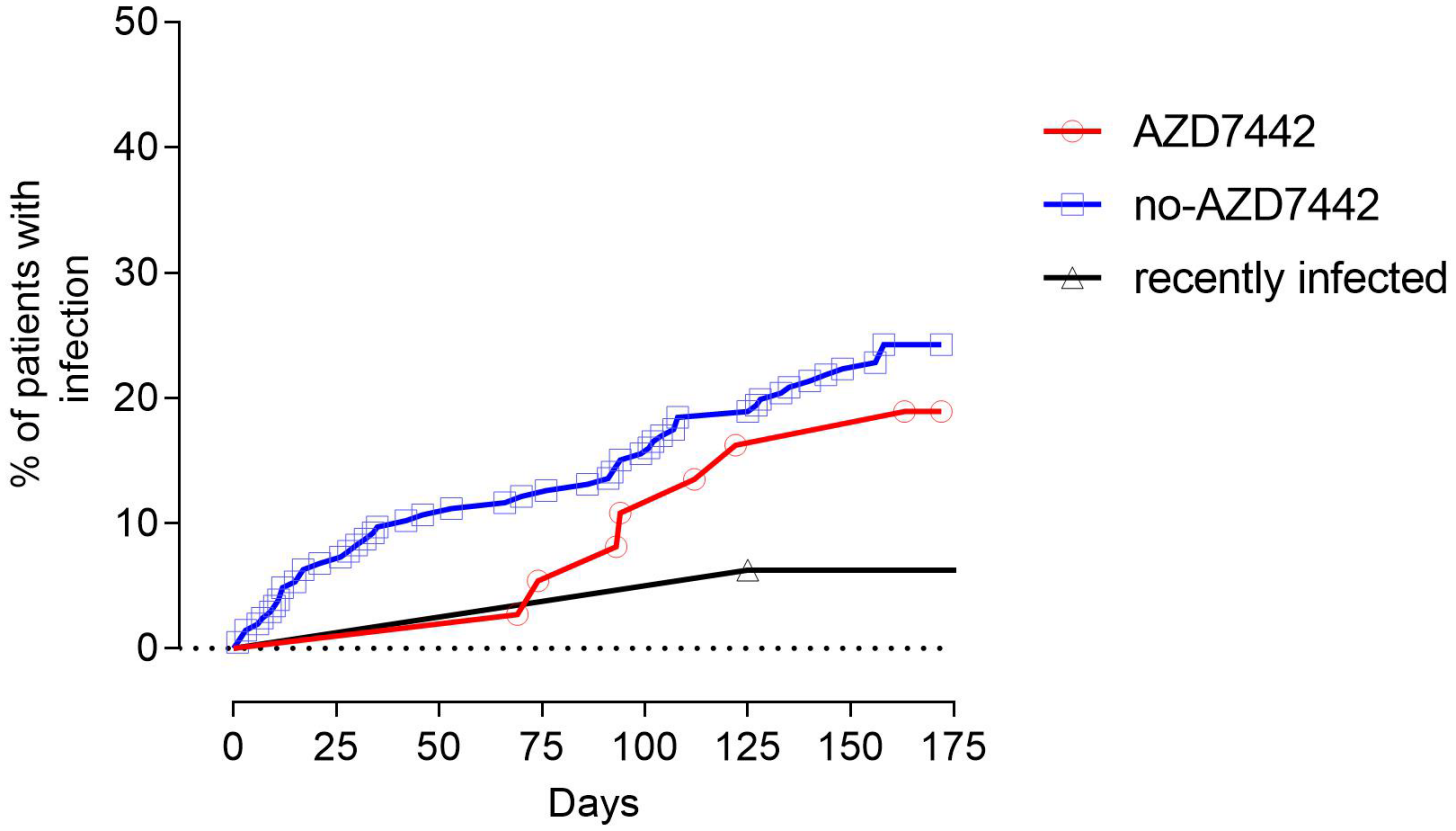
The proportion of patients being infected by SARS-CoV-2 in the study groups. Patients treated with AZD7442 as pre-exposure prophylaxis are represented as a red line, and patients who were not treated with AZD7442 are represented as a blue line. Patients who were infected in the three months preceding the enrolment are separately analysed and represented as a black line.

A total of 60 SARS-CoV-2 infections occurred during the study period. Six out of thirty-three (18.2%) patients who received AZD7442 were infected with SARS-CoV-2 compared to 54/170 (31.8%) patients who did not receive AZD7442 (p = 0.048). Infections occurred respectively after a median time of 167.1 days (95%CI 154.9-179.3) and 148 days (95%CI 138.9-157.33) from the enrolment. The risk of SARS-CoV-2 infection was reduced by 85% among those who received the PrEP at a median follow-up of 90 days from treatment administration (hazard risk [HR] 0.15; 95%CI 0.14-0.96; log-rank p=0.036). The comparison was not significant at the end of the study period (HR 0.6; 95%CI 0.33-0.1.84; log-rank p=0.278) (**Figure 3**). No patient who had received prophylaxis became infected in the first 60 days, while those who had not received prophylaxis continued to become infected with a constant trend over time. To note, when considering only SARS-CoV-2 naive patients, the AZD7442 group had a lower risk for infection than the no-AZD7442 group at a median follow-up of 90 days (HR 0.20, 95%CI 0.07-0.56, log-rank p=0.049, **Supplementary Figure 2**). Time of viral shedding did not differ in the AZD7442 and no-AZD7442 groups (19 days, IQR: 10-28 vs. 13 days, IQR: 7-20, p=0.614). At univariate analysis, age was positively associated with SARS-CoV-2 infection (OR 4,841, 95%CI 0,07-9,61, p= 0.047), while a prior episode of SARS-CoV-2 infection was found to be protective (OR 0.09 95%CI 0,03-0,32, p<0.0001). Gender, lymphocyte counts, IgA serum levels, IEI comorbidities, complicated phenotype, and the number of doses of COVID-19 vaccine were not associated with infection risk (**Supplementary Table 2**). Immunophenotype of infected patients revealed a lower frequency of MBC cells in the AZD7442 group compared to the no-AZD7442 group (median 7.5%, IQR 26.5-48.5 vs. 1.0%, IQR 4.2-16.0, p=0.040) (**Supplementary Figure 3**), suggesting that PreEP was ineffective mainly in patients with severely impaired memory B function.

The recently infected group was 89% and 92% less likely to have a SARS-CoV-2 infection compared to the AZD7442 and the no-AZD7442 group (HR 0.11; 95%CI 0.03-0.61; log-rank p=0.011 and HR 0.08; 95%CI 0.16-0.64; log-rank p=0.001, **Figure 3**), as a single patient was infected during the study.

### Course of COVID-19

None of the patients was hospitalized for COVID-19, had severe SARS-CoV-2 infection, or died during the study. A mild symptomatic infection was observed in 29% of patients of the no-AZD7442 group and in 18% of the AZD7442 group (p=0.500). The risk of symptomatic infection was reduced among the AZD7442 group within the first 90 days (HR 0.18; 95%CI 0.14-1.05; log-rank p=0.049). Univariate analysis identified low lymphocyte counts as a risk factor for developing symptomatic COVID-19 (**Supplementary Table 3**). SARS-CoV-2 infection required treatments in 33% of patients in the AZD7442 group (antivirals only) and in 56% of patients in the no-AZD7442 group (antivirals and monoclonal antibodies).

### anti-Spike IgG serum levels in AZD7442 treated patients

**Figure 4:**
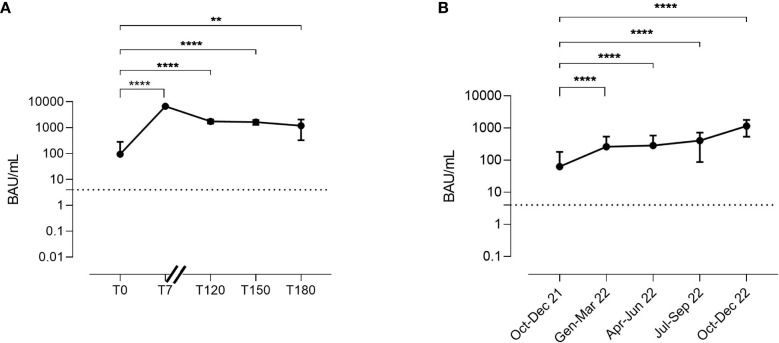
Kinetics of anti-Spike IgG serum levels in the AZD7442 recipients **(A)**. Anti-Spike IgG serum levels in patients under IgRT over time **(B)**. Anti-Spike IgG was randomly measured over 15 months in plasma samples collected from patients just before intravenous administration. The lower and the upper detection limits of the assay are represented by the dashed line. Points indicate medians, bars indicate interquartile ranges. Non-parametric Mann–Whitney t-test calculated by the first and the following time points was used to evaluate the statistical significance. Two-tailed P value significance is shown as ** p<0.01 and ****p<0.0001. Number of samples tested: **(A)**, T0 n=30, T7 n=30, T120 n=25; T150 n=15, T180 n=8; **(B)**, Oct-Dec 21 n=53; Jen-Mar 22 n=35, Apr-Jun 22 n=15, Oct-Dec 22 n=22. BAU, binding antibody unit; T, time point.

In the AZD7442 group, median anti-Spike IgG serum levels at enrolment were 18.5 BAU/mL (IQR: 3.7-77.7). Seven days after AZD7442 administration (T7), IgG median levels were 6780 BAU/mL (IQR: 6305-7075) and they remained high and at T120 (1695 BAU/mL, IQR: 1548-2080), at T150 (1580 BAU/mL, IQR 1345-1935), and at T180 (1120 BAU/mL, IQR 754-2013) (**Figure 4A**).

### SARS-CoV-2 IgG antibodies and polyclonal immunoglobulin administration

As IgRT might contribute to the passive transfer of anti-Spike IgG, we randomly measured anti spike IgG serum levels in plasma samples of patients who did not receive AZD7442 as therapy or as prophylaxis. To avoid bias, we excluded patients infected in the previous six months or vaccinated in the previous four weeks. Anti-Spike IgG serum levels increased over time, from a median level of 7.5 BAU/mL (IQR: 0-101.1) recorded in the trimester October-December 2021 to a median level of 1175 BAU/mL (IQR: 663-1728) recorded in the trimester October-December 2022 (p<0.0001) (**Figure 4B**), showing the progressive increase of the anti-Spike IgG concentration in the polyclonal immunoglobulin brands administered as IgRT.

## Discussion

Since the beginning of the COVID-19 pandemic, protective strategies for patients with IEI have been continuously adapted to the dynamics of the epidemic and updated based on recent knowledge and therapeutic options available (7). The period starting from July 2022 proved to be critical due to the emergence of new viral variants (29), the possible waning of vaccine-induced immunity (30), and the decrease of the neutralising power of the available SARS-COV-2 mAbs (31–33). Consequently, bivalent booster doses of the COVID-19 vaccine targeting the Omicron sub-lineages BA.1 (34) and BA.4/BA.5 (35) were approved for fragile subjects. In addition, mAbs were approved firstly as early treatment and later as prophylaxis for vulnerable patients (22, 23, 36). However, two large real-life studies enrolling immunocompromised patients reported a low rate of infections and severe illnesses in participants who received prophylactic AZD7442 (24, 37). Despite being immunised with at least three doses of mRNA vaccine, most patients with primary antibody deficiencies (PAD) showed suboptimal responses to immunisation as they did not produce specific antibodies or generate high-affinity spike-specific B nor RBD+ B cells (13, 28, 38). Thus, the possibility to administer a specific prophylactic treatment was a significant opportunity.

Here we conducted the largest study on PAD patients treated by intramuscular AZD7442 as PrEP. In this real-life setting, patients who received prophylaxis showed a reduced overall risk of SARS-CoV-2 infection and symptomatic infection within the first 90 days post-administration. Differently, those who had not received AZD7442 became infected with a constant trend during the study. PreEP was ineffective mainly in patients with severely impaired memory B function. Increasing age was confirmed to be associated with the risk of infection (39), while previous episodes of SARS-CoV-2 infection had a protective effect.

A significant limitation of the present study is the small number of patients receiving the PrEP compared to the non-treatment group, leading to a potential bias. Despite no selection performed at enrolment, patients in the AZD7442 group were found to have very low IgA serum levels and more defective post-immunization serological responses, two well-known risk factors for SARS-CoV-2 and other respiratory infections (10, 28, 40). We hypothesised that due to a more severe disease, these patients have been more likely to accept PrEP. Differences in baseline characteristics might have led to a potential interpretation bias, reducing the magnitudes of the positive effect of the AZD7442 prophylaxis.

The beneficial effect of mAbs given pre-exposure prophylaxis was reduced three months post- AZD7442 administration, possibly due to the appearance of the new VOCs. We did not have data to support this hypothesis since we did not type SARS-CoV-2 in the positive swabs or measure the neutralising power of the AZD7442. Nevertheless, normative data reported that the prevalent VOCs in Italy in November-December 2022 were the Omicron B.1.1.529 variants (41), towards which AZD7442 had a reduced efficacy (42).

The reduced protective effect of the PrEP observed over time did not match with kinetic data that shows the persistence of high antibody titers maintained high due to the regular administration of polyvalent immunoglobulins. However, the contribution of IgRT on protection might be low since polyvalent immunoglobulins were obtained from plasma collected at least six months before the study time from donors that possibly did not have developed specific antibodies.

In summary, at the end of the observation period, we had two potential passive prophylaxis strategies: SARS-CoV-2 mAbs and polyvalent immunoglobulins. However, the efficacy of both strategies needs to be constantly re-evaluated by real-world studies, considering that emerging epitope mutations in the viral genome might result in increased antibodies immune evasion (43).

To note, the quantification of specific antibody serum levels can offer partial information about the protection status since antibodies cannot be assumed as the only absolute correlate of protection. The complex immune response to SARS-CoV-2 leading to short- and long-lasting immune memory is mediated by specific antibodies, memory T- and B-cells and plasma cells (28, 44, 45). The combined analysis of Spike-specific IgG and Spike-specific B cells shows that patients with primary antibody defects have different degrees of immune impairment of responses towards SARS-CoV-2 (28). Should we target the therapeutic strategy accordingly?

Some Authors have suggested the futility of continuing to vaccinate non-responder patients (39). Since none of the patients enrolled in the study required hospitalisation and none died, our approach in the coming months will not change until possible new strategies. Precaution measures, immunisation and antivirals will continue to represent the best date option to protect vulnerable patients with primary antibody defects against COVID-19. Finally, it should be mentioned that the strategy to administer monoclonal antibodies and IgRT as therapy or prophylaxis contributes to protection thanks to their neutralising activity but also to their capacity to prime the other facets of the immune response due to their Fc-mediated immunomodulatory activities (46, 47).

## Data availability statement

The raw data supporting the conclusions of this article will be made available by the authors, without undue reservation.

## Ethics statement

The studies involving humans were approved by Ethical Committee of the Sapienza University of Rome. The studies were conducted in accordance with the local legislation and institutional requirements. The participants provided their written informed consent to participate in this study.

## Author contributions

Conceptualization FP, IQ, and GG: methodology, LC, PR, and AN: formal analysis, FP: investigation, RC, EM: experiments, CM, AP, MC, GN, ES: resources, IQ: data curation, FP: writing original draft preparation, FP and IQ: writing, review and editing, project administration, GG: funding acquisition, IQ, RC. All authors contributed to the article and approved the submitted version.
