# Supplementary material for: SARS-CoV-2 pre-exposure prophylaxis with tixagevimab/cilgavimab (AZD7442) provides protection in inborn errors of immunity with antibody defects: a real-world experience
Source: Front Immunol. 2023 Oct 26;14:1249462. doi: 10.3389/fimmu.2023.1249462 (PMC10639167; doi:10.3389/fimmu.2023.1249462)
Supplement: Supplementary file 1 [file DataSheet_1.docx]

***Supplementary Material***

**SARS-CoV-2 pre-exposure prophylaxis with tixagevimab/cilgavimab (AZD7442) provides protection in Inborn Errors of Immunity with Antibody Defects: a real-world experience.**

**Federica Pulvirenti, MD, PhD, Giulia Garzi, MD, Eleonora Sculco, MD, Maddalena Sciannamea, MD, Cinzia Milito, MD, PhD, Anna Napoli, MD, Lilia Cinti, BD, Piergiorgio Roberto, BD, Alessandra Punziano, MD, Maria Carrabba, MD, PhD, Eva Piano Mortari, Rita Carsetti, Guido Antonelli, MD, PhD, Isabella Quinti, MD, PhD*.**

**Correspondence:** Isabella Quinti, Department of Molecular Medicine, Viale Regina Elena 291, 00161, Rome, Italy. Telephone number: +300649972007. Email address: isabella.quinti@uniroma1.it.

**Supplementary Tables**

**Supplementary Table 1**. Characteristics of patients recently infected by SARS-CoV-2 within three months before the enrollment.

|  | Recently infected  n= 35 | p value^1^ | p value^2^ |
| --- | --- | --- | --- |
| Age (years), median (IQR) | 54 (41-66) | 0.498 | 0.865 |
| Gender (female), n (%) | 21 (61.8) | 0.199 | 0.397 |
| IgA (mg/dL), median (IQR) | 11 (2-59) | 0.106 | 0.005 |
| Residual IgG (mg/dL), median (IQR) | 621 (605-670) | 0.503 | 0.801 |
| IEI Diagnosis |  |  |  |
| CVID, n (%) | 22 (62.9) | 0.065 | 0.082 |
| XLA, n (%) | 2 (5.7) | 0.801 | 0.590 |
| Good syndrome, n (%) | 1 (2.9) | 0.860 | 0.966 |
| UAD, n (%) | 9 (25.7) | 0.003 | 0.072 |
| Others n (%) | 1 (2.9) | 0.353 | 0.966 |
| Lymphocytes count, median (IQR) | 1620 (1160-2040) | 0.908 | 0.171 |
| CD3+CD4+ (cell/mm3), median (IQR) | 472 (402-784) | 0.799 | 0.366 |
| CD19+ (cell/mm3), median (IQR) | 83.9 (8.2-250.6) | 0.641 | 0.381 |
| MBC (CD19+CD27, cell/mm3), median (IQR) | 21.2 (2.4-39.9) | 0.774 | 0.952 |
| Complicated phenotype, n (%) | 10 (30) | 0.078 | 0.016 |
| COPD, n (%) | 23 (46.7) | <0.0001 | 0.068 |
| SARS-COV-2 vaccine doses >=3, n (%) | 35 (100) | 0.018 | 0.068 |

Abbreviation: IEI inborn errors of immunity, CVID common variable immunodeficiency, XLA X linked agammaglobulinemia, UAD undefined antibody deficiency, COPD Chronic Pulmonary Disease, COVID-19 coronavirus disease 2019, IQR interquartile range, MBC memory B cells, SARS-CoV-2 severe acute respiratory syndrome coronavirus 2.

^1^comparison with No-AZD7442 group; ^2^comparison with AZD7442 group.

**Supplementary Table 2.** Univariate analysis of patient’s characteristics associated with SARS-CoV-2 infection.

|  | Not infected  n= 156 | SARS-Cov-2 infection  n=59 | p value |
| --- | --- | --- | --- |
| Age (years), median (IQR) | 49 (36-59) | 54 (45-64) | 0.047 |
| Gender (female), n (%) | 69 (44.2) | 32 (54.2) | 0.439 |
| Serum IgA (mg/dL), median (IQR) | 2 (1-22) | 5 (2-13) | 0.439 |
| Lymphocytes count, median (IQR) | 1445 (1130-2020) | 1460 (930-1890) | 0.146 |
| CD3+CD4+ (cell/mm3), median (IQR) | 516 (400-728) | 578 (395-859) | 0.797 |
| CD19+ (cell/mm3), median (IQR) | 52.5 (21.9-161.1) | 57.6 (11-134.5) | 0.635 |
| CD19+ CD27+ (% of CD19+), median (IQR) | 22.2 (5.9-45) | 22.6 (8-41) | 0.468 |
| Complicated phenotype, n (%) | 59 (47.2) | 23 (47.9) | 0.804 |
| COPD, n (%) | 52 (33.6) | 24 (41.7) | 0.322 |
| Prior episode of COVID-19, n (%) | 56 (36.1) | 3 (5.1) | <0.0001 |
| Vaccinated (>=3 doses), n (%) | 134 (86.3) | 52 (88.1) | 0.668 |
| Last vaccine dose within 6 months | 79 (50.7) | 25 (43.2) | 0.279 |

Abbreviation: IEI inborn errors of immunity, CVID common variable immunodeficiency, XLA X linked agammaglobulinemia, UAD undefined antibody deficiency, COPD Chronic Pulmonary Disease, COVID-19 coronavirus disease 2019, IQR interquartile range, SARS-CoV-2 severe acute respiratory syndrome coronavirus 2.

**Supplementary Table 3.** Univariate analysis of patient’s characteristics associated with symptomatic SARS-CoV-2 infection.

|  | Symptomatic infections  n=42 | Asymptomatic infections  n=10 | p value |
| --- | --- | --- | --- |
| Age (years), median (IQR) | 52 (43-58) | 50 (25-55) | 0.179 |
| Gender (female), n (%) | 21 (50) | 3 (30) | 0.254 |
| Serum IgA (mg/dL), median (IQR) | 3 (2-20) | 12 (2-43) | 0.520 |
| Lymphocytes count, median (IQR) | 1745 (1280-2280) | 1070 (990-1380) | 0.034 |
| Complicated phenotype, n (%) | 19 (45.2) | 2 (20) | 0.144 |
| COPD, n (%) | 10 (23.8) | 4 (40) | 0.299 |
| Prior episode of COVID-19, n (%) | 14 (33.3) | 2 (20) | 0.412 |
| Vaccinated (>=3 doses), n (%) | 34 (80.1) | 8 (80) | 0.945 |
| Last vaccine dose within 6 months | 26 (61.9) | 5 (50) | 0.490 |

Abbreviation: COPD Chronic Pulmonary Disease, COVID-19 coronavirus disease 2019, IQR interquartile range, SARS-CoV-2 severe acute respiratory syndrome coronavirus 2.

**Supplementary Figure 1.** Gating strategy. (A) FACS plots depict the gating strategy for the identification of total (CD19+CD24+CD27+), IgM+ and IgM- MBCs, in a representative HCW. Low (S+) and high (S++) affinity spike-specific MBCs are shown. (B) IgM+, IgM- and RBD+ MBCs among S+ and S++. IgM expression on receptor binding domain (RBD+) MBCs is shown.


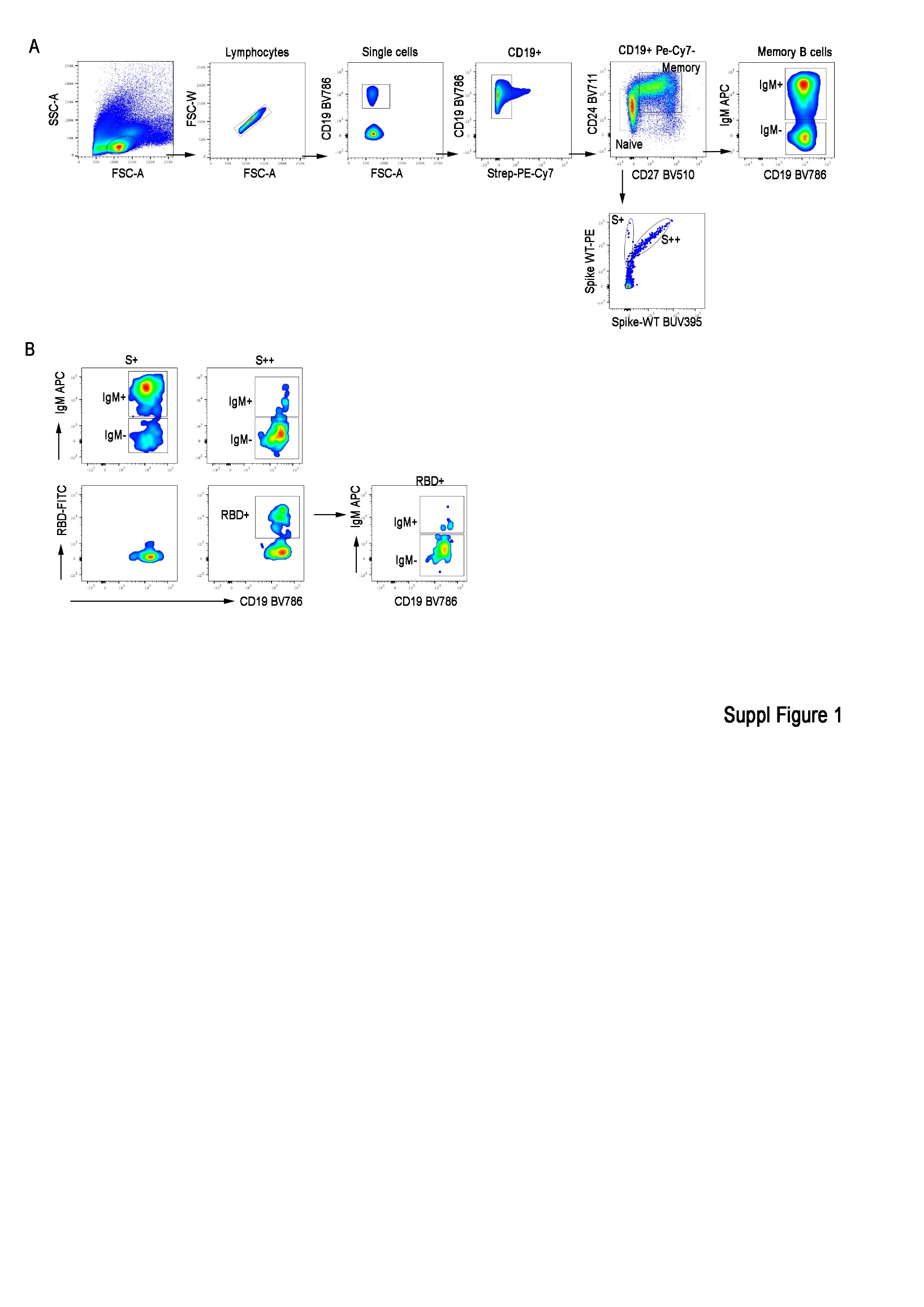


**Supplementary Figure 2.** Patients infected by SARS-CoV-2 in the AZD7442 group (red line) and in the no-AZD7442 (blue line) stratified for the entry condition of being COVID-19 naive. The difference between the two groups is expressed as Log-rank (the last significant comparison was reported). Red dots and blue squares represent participants infected in the AZD7442 and in the no-AZD744 group, respectively.

**
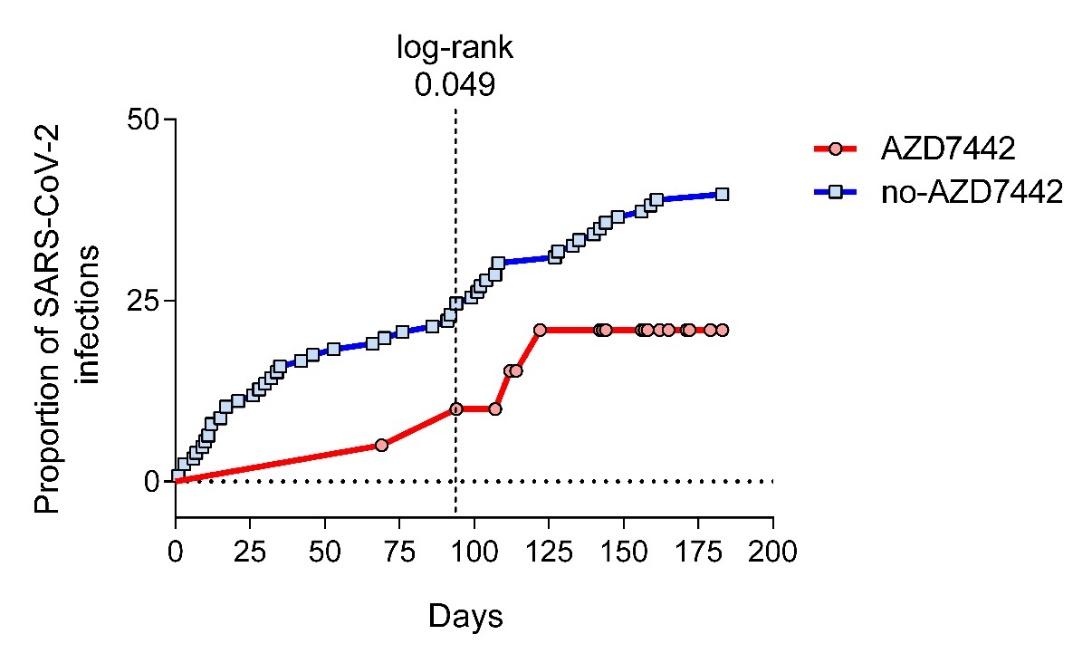
**

**Supplementary Figure 3.** The proportion of MBC (CD19+CD27+) recorded in patients infected or not by SARS-CoV-2 in the AZD7442 (red histograms) and in the no-AZD7442 group (blue histograms). Histograms indicated median. Non-parametric Mann–Whitney t-test was used to evaluate statistical significance. Two-tailed P value significances are shown as *p< 0.05. Abbreviations: MBC memory B cells, COVID-19 coronavirus 19 disease.
